# Re-evaluating experimental validation in the Big Data Era: a conceptual argument

**DOI:** 10.1186/s13059-021-02292-4

**Published:** 2021-02-24

**Authors:** Mohieddin Jafari, Yuanfang Guan, David C. Wedge, Naser Ansari-Pour

**Affiliations:** 1 Faculty of Medicine, University of Helsinki, Helsinki, Finland; 2 Department of Computational Medicine and Bioinformatics, University of Michigan, Ann Arbor, MI 48109 USA; 3 Manchester Cancer Research Centre, The University of Manchester, Manchester, M20 4GJ UK; 4 Big Data Institute, Nuffield Department of Medicine, University of Oxford, Oxford, OX3 7LF UK

Many researchers who work in fields such as bioinformatics and biomathematics, will have, at some point, come across the well-known query of whether they have ‘experimentally validated’ their results. This question, which is predominantly raised by people outside the computational field, is often asked with cynicism or at best suspicion towards results obtained from computational, mathematical or statistical models and more generally theoretical reasoning applied to empirical observations in an automated fashion. We argue here that the combined use of orthogonal sets of computational and experimental methods within a scientific study can increase confidence in its findings and that the use of the term ‘experimental validation’ is a hindrance to this endeavour.

Many misunderstandings—scientific or otherwise—stem from the presence of a language barrier. We do not here refer to differences in the set of words or expressions used by scientists in different specialties, but rather to the enlistment of terms in common general usage to denote a precise scientific concept. Take for example the term ‘normal distribution’, which is used extensively when making statistical inferences in various empirical studies [1]. Although this term is often understood correctly as a statistical concept, it can also be misconstrued due to the connotations in everyday use of the term ‘normal’, i.e. natural, standard, correct or even healthy [2, 3].

Similar misunderstandings arise with the phrase *experimental validation*. Within the field of computational biology, ‘experimental validation’ refers to the process of reproducing a scientific finding obtained using computational methods by performing investigations that do not rely on the extensive use of computational resources. This process involves the accumulation of additional evidence that lend support to the conclusions of the computational study. However, as with the term ‘normal’, the term ‘validation’ carries with it connotations from everyday usage such as ‘prove’, ‘demonstrate’, ‘authenticate’ or even ‘legitimise’. We would argue that these associations are a hindrance to scientific understanding and that the term *experimental validation* would be better replaced with alternative terms, such as ‘experimental calibration’ or ‘experimental corroboration’.

## Theoretical modelling

Consider a computational model that aims to explain a biological phenomenon such as tumour growth; how a cancer initiates, progresses and expands; and how it can be halted by the use of a therapeutic compound. The question often asked is whether the results of this model should be assessed, e.g. in vitro with a cell line assay for it to be of any merit. In other words, is this validation step critical to claim that the computational model works correctly?

To answer this, we should revisit the concept of a computational model. Simply put, a computational model comprises a mathematical framework built upon assumptions, which are themselves derived from a series of empirical observations. In the above-mentioned example, we know that cancer is a genetic disease with abnormal cell proliferation where the number of cells is increased by one upon each cell division. If our computational model fails to accurately estimate the number of cancer cells after *n* divisions, this deviation does not originate from the exponential function but rather from our assumptions or over-simplicity of the model. For instance, we did not include other variables in our model such as nutrient levels or interaction of the tumour with the immune system. In fact, it is essential to have some level of empirically-based knowledge a priori for model reconstruction. However, the critical point is that the computational model itself does not require validation, as it is merely a logical system for deducing more complex features from a priori data [4]. Previously acquired empirical knowledge has an important role to play in tuning the parameters of a model, and model parameters may be adjusted to fit experimental data optimally. The role of experimental data in this scenario would be better described, rather than ‘validation’, as ‘calibration’. We should also note that in some instances experimental calibration is not appropriate to test the accuracy of a computational model when the ground truth is unknown. For example, early cancer genome studies predicted the titin gene (*TTN*) as a cancer driver based on a selection pressure model incorporating the non-synonymous to synonymous mutation ratio [5]. Hitherto, due to having the longest coding sequence in the human genome [6], *TTN* has been found as a recurrently hit gene in many cohorts across multiple tumour types and its frequent presence is highly likely to be ‘validated’ in further independent cohorts. However, an improved driver model, which adjusts for background mutation rate [7], no longer identifies *TTN* as a cancer driver but rather more likely a footprint of somatic mutation accumulation.

## Data-driven inference

The recent advent of high-throughput technologies has enabled the generation of awe-inspiring amounts of biological data, especially in the realm of omics, and developing accurate methods and models to analyse and interpret data is as crucial as the data generation itself. In other words, big data has changed the way we deal with biological data simply because of its size. For example, it would be infeasible to look at DNA sequencing chromatograms for millions of reads to analyse the genomic sequence of a sample, and therefore sophisticated methods and pipelines have been developed to deal with signal reading, alignment and variant calling. It is thus important to remember that computational methods have been developed out of necessity to deal with such big data, rather than as a replacement for experimentation, which remains core to biology in the new era.

A question often raised in different academic settings after a biological inference has been made using computational analysis of high-throughput biological data, is whether it is essential to undertake additional low-throughput experiments to validate the inference?

While low-throughput gold standard methods (such as Sanger dideoxy sequencing or Western blotting) may seem more tangible to many, it does not necessarily follow that such methods are more reliable or robust than high-throughput methods, particularly given the inherently variable nature of empirical data. For this reason, the performance of an experimental study that represents an orthogonal method for partially reproducing the results of a computational biology study is more appropriately described as ‘corroboration’ than ‘validation’. We illustrate this with a series of examples as follows. We also argue that when dealing with orthogonal approaches, more confidence should be placed in inferences derived from data from higher throughput or higher resolution experiments.

### Copy number aberration calling (WGS vs FISH)

Copy number aberration (CNA) is a hallmark of virtually all cancer types. Its accurate estimation is not only key in estimating other features of a given tumour such as genomic instability and purity, but, at the functional level, it is also essential in estimating gene dosage and assessing the two-hit hypothesis (bi-allelic inactivation) for any given gene. CNA is nowadays called at the genome-wide level based on whole-genome sequencing (WGS) data of the tumour sample and its matched normal pair. The most recently developed methods can not only detect total copy number, but also allele-specific copy number, and can distinguish CNAs found in all tumour cells (‘clonal’ events) from those found in a subset of cells (‘subclonal’) [8, 9]. These methods have the resolution to detect smaller CNAs than previous methods such as SNP arrays and array comparative genomic hybridisation (aCGH) since they utilise read counts from every base-pair within the genome. Karyotyping and fluorescent in-situ hybridisation (FISH) of a number of cells (typically ~ 20–100 cells) have served as gold standard methods to detect CNAs in a tumour. FISH has superior resolution to karyotyping [10], but usually utilises one or a few locus/chromosome-specific probes to extrapolate the absence/presence and count of an entire chromosome, while WGS-based CNA calling is based on signals from thousands of SNPs in a region. Further, FISH-based analysis requires a trained eye to distinguish a hybridisation signal from the background colour noise and is somewhat subjective, while the WGS-based computational methods are quantitative, and typically use statistical thresholds to call CNAs. While FISH has advantages in certain scenarios, such as the detection of whole-genome duplicated samples, it is not clear that results from FISH are more reliable than those from WGS, and it provides lower resolution of subclonal and sub-chromosome arm size events. For this reason, we encourage the field to embrace alternative methods for corroborating CNA calls, such as the use of low-depth WGS of thousands of single cells [11].

### Mutation calling (WGS/WES vs Sanger)

One of the core analyses of WES/WGS is variant calling using germline (e.g. Platypus [12]) or somatic (e.g. MuTect [13]) pipelines to identify aetiological variants in disease predisposition or initiation/progression respectively. If novel variants or genes are identified, there is commonly a desire to replicate this finding. The gold standard for variant detection is Sanger dideoxy sequencing. However, this method cannot reliably detect variants with variant allele frequency (VAF) below ~ 0.5. In the case of mosaicism [11] at the germline level and low-purity clonal variants or high-purity subclonal variants at the somatic level [14] Sanger sequencing will not detect variants that have been detected by relatively high coverage WGS and higher coverage WES experiments. A more appropriate replication would be high-depth targeted sequencing of detected loci of interest. This method not only has greater power to detect the candidate variants, but can also give more precise VAF estimates, and is more readily applied to a much larger number of variants.

### Differential protein expression (mass spectrometry vs Western blot/ELISA)

Mass spectrometry (MS) has revolutionised proteomics by delivering robust, accurate and reproducible protein detection in recent years. The gold standard in the field, however, remains the western blotting assay which is a non- or semi-quantitative method for the detection of specific proteins in complex biological samples based on the specificity of select antibodies. Given the fine detail of MS and, crucially, the higher number of data points in this method, the presence/absence of a peptide/protein can be called with much higher confidence [15]. For instance, in a complex extract of a tumour sample, compare MS results pertaining to a protein based on more than five peptides that cover ~ 30% of the protein sequence (with an *E* value < 10^−10^) with the results of three replicates of western blotting by using an antibody with a linear epitope which has less than 1% coverage. It is clear that more confidence can be placed in the reliability of MS results. In addition, it is worthy of note that antibodies for Western Blot/ELISA are not available for all identified proteins and, even if available, they may not have the expected efficiency due to the high rate of non-silent somatic mutations in cancer cells. Consistent with this, it was recently argued that there should be a reprioritisation where MS supersedes western blotting due to its higher resolution [16].

### Differentially expressed genes (RNA-seq vs RT-qPCR)

Reprioritisation of experimental methods has also occurred in transcriptomic studies [17–21]. Whole-transcriptome RNA-seq is a comprehensive approach for the identification of transcriptionally stable genes compared with reverse transcription-quantitative PCR (RT-qPCR). The high coverage of RNA-seq analysis of an intricate RNA pool of cancer cells also enables the identification of transcripts within the sample to nucleotide-level resolution in a sequence-agnostic fashion thus allowing detection of novel expressed genes [22]. RNA-seq techniques have also allowed further exploration of the complexity and heterogeneity of tumours by single-cell transcriptomics of tumour samples, which is not feasible with RT-qPCR.

**Fig. 1:**
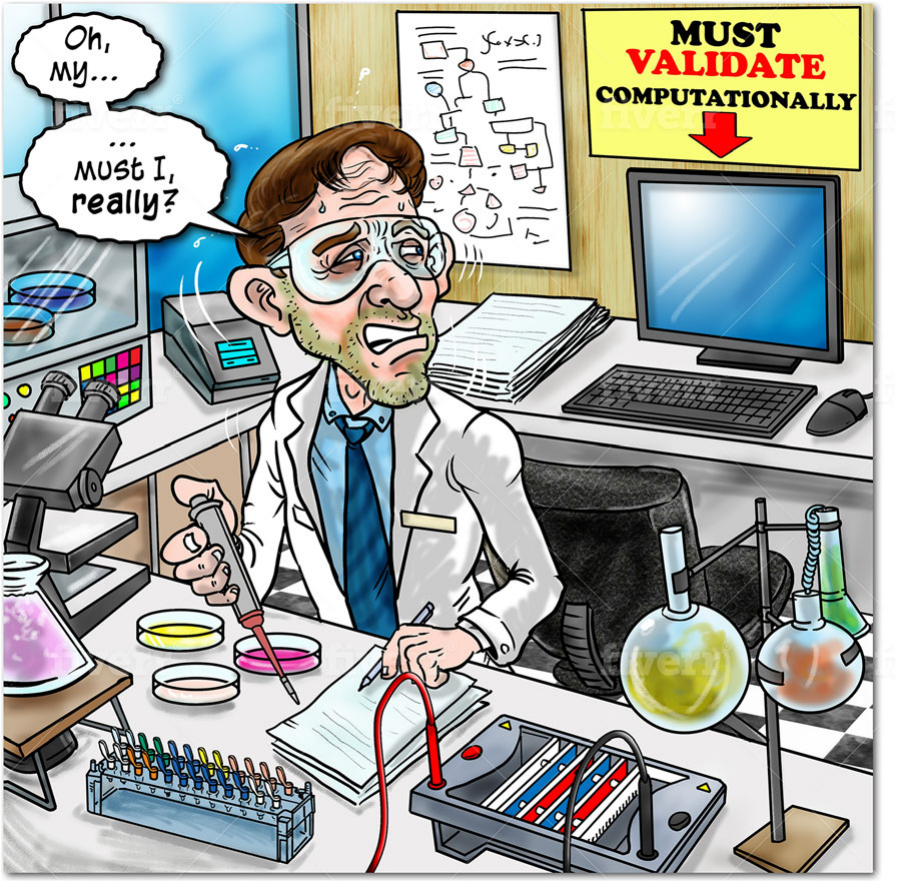
All research in wet- and dry-lab biology use computational methods that examine strength of evidence [24]. Everybody starts from the lab with original data generation or data collection, and it only diverges when we try to analyse and make inferences. We argue that ‘computational validation’ is undertaken in all experimental and computational studies. For instance, replication of experiments in a case-control setting is in itself inherently a computational validation since we compare mean ± SD or any other computationally-derived summary metric between cases and controls to distinguish signal from noise. Therefore, ‘computational validation’ is a term that should be publicised along with ‘experimental validation’, to scientifically reproduce findings from experimental as well as computational biology. Illustration by Ricky Castillo

In conclusion, we strongly support multidisciplinary science that integrates computational and experimental biology studies. We believe that the use of the phrase ‘experimental validation’ is hindering rather than enabling such research, by preventing objective evaluation of the relative strengths of high-throughput and low-throughput methods, and we encourage the use of less value-laden terms such as ‘experimental calibration’ or ‘experimental corroboration’. As in experimental biology, computational biology requires an understanding of the limitations of the existing methods, which generally is acquired from years spent working with big data [23]. It is essential that computational scientists convey the intricacies of their methods to non-computational colleagues to enable appropriate interpretation and evaluation of results, especially when multiple tools are available for the same analysis. Evaluating the underlying assumptions of computational models often represents a superior method of evaluation than conducting further empirical studies. Finally, we encourage all scientists to evaluate all empirical evidence, whether from high-throughput or low-throughput experiments, in a balanced manner, even if this means putting more faith in modern high-throughput experiments than older techniques commonly regarded as ‘gold standard’. We thus envision a world where ‘experimental’ scientists are requested to do ‘computational validation’ in the same manner that ‘computational’ scientists are currently asked to do experimental validation (Fig. 1).
